# The ubiquitin E3 ligase APC/C^Cdc20^ mediates mitotic degradation of OGT

**DOI:** 10.1016/j.jbc.2024.107448

**Published:** 2024-06-04

**Authors:** Li Meng, Rui Dong, Weixiao Mi, Ke Qin, Kunfu Ouyang, Jianwei Sun, Jing Li

**Affiliations:** 1 Beijing Key Laboratory of DNA Damage Response and College of Life Sciences, Capital Normal University, Beijing, China; 2 State Key Laboratory for Conservation and Utilization of Bio-Resources in Yunnan, Yunnan Key Laboratory of Cell Metabolism and Diseases, Center for Life Sciences, School of Life Sciences, Yunnan University, Kunming, China; 3 College of Chemistry and Molecular Engineering, Beijing National Laboratory for Molecular Sciences, Peking-Tsinghua Center for Life Sciences, Synthetic and Functional Biomolecules Center, and Key Laboratory of Bioorganic Chemistry and Molecular Engineering of Ministry of Education, Peking University, Beijing, China; 4 Department of Cardiovascular Surgery, Peking University Shenzhen Hospital, Shenzhen, China

**Keywords:** OGT, ubiquitination, uterine carcinoma, APC/C^Cdc20^, cell cycle

## Abstract

O-linked β-N-acetylglucosamine (O-GlcNAc) transferase (OGT) is the sole enzyme that catalyzes all O-GlcNAcylation reactions intracellularly. Previous investigations have found that OGT levels oscillate during the cell division process. Specifically, OGT abundance is downregulated during mitosis, but the underlying mechanism is lacking. Here we demonstrate that OGT is ubiquitinated by the ubiquitin E3 ligase, anaphase promoting complex/cyclosome (APC/C)-cell division cycle 20 (Cdc20). We show that APC/C^Cdc20^ interacts with OGT through a conserved destruction box (D-box): Arg-351/Leu-354, the abrogation of which stabilizes OGT. As APC/C^Cdc20^-substrate binding is often preceded by a priming ubiquitination event, we also used mass spectrometry and mapped OGT Lys-352 to be a ubiquitination site, which is a prerequisite for OGT association with APC/C subunits. Interestingly, in The Cancer Genome Atlas, R351C is a uterine carcinoma mutant, suggesting that mutations of the D-box are linked with tumorigenesis. Paradoxically, we found that both R351C and the D-box mutants (R351A/L354A) inhibit uterine carcinoma in mouse xenograft models, probably due to impaired cell division and proliferation. In sum, we propose a model where OGT Lys-352 ubiquitination primes its binding with APC/C, and then APC/C^Cdc20^ partners with OGT through the D-box for its mitotic destruction. Our work not only highlights the key mechanism that regulates OGT during the cell cycle, but also reveals the mutual coordination between glycosylation and the cell division machinery.

The O-linked β-N-acetylglucosamine (O-GlcNAc) transferase (OGT) utilizes the Uridine diphosphate (UDP)-GlcNAc as the donor and installs the GlcNAc moiety to the Ser/Thr residues of intracellular proteins (1, 2). The role of OGT during the cell division process has long been appreciated (3, 4, 5, 6, 7), and myriad OGT substrates have been identified in regulating the mitotic spindle dynamics (8, 9, 10), centrosome disjunction (11, 12), chromosome segregation (13), midbody (14) and cytokinesis (4, 15, 16).

Intriguingly, OGT itself is subject to the regulation of the cell cycle machinery. Upon mitotic entry, the overall O-GlcNAc levels decrease (15, 17), probably due to a decrease in both the OGT protein level and mRNA level (18). OGT also re-localizes to the mitotic spindle and its overproduction results in chromosome bridges (4, 18). During cytokinesis, work from our lab demonstrated that OGT is phosphorylated by the checkpoint kinase 1 (Chk1) kinase at Ser-20, and the phospho-specific pS20 antibody localizes to the midbody (14). OGT Ser-20 is also phosphorylated by the calcium/calmodulin-dependent kinase II (CaMKII) (19). In *Drosophila*, mouse embryonic stem cells and mouse embryonic fibroblasts, Chk1-mediated pS20 of OGT is important for not only fly gut homeostasis but also DNA damage response in mouse cells (20), suggesting that studying modifications on OGT itself provides valuable insights into understanding its function (21).

The ubiquitin E3 ligase anaphase-promoting complex/cyclosome (APC/C) orchestrates a series of mitotic events to ensure the faithful segregation of sister chromatids (22, 23, 24). Structurally, it is composed of 14 distinct subunits, comprising the platform, the catalytic core and the tetratricopeptide repeat (TPR). The APC/C remains largely inactive until it cooperates with one of its two co-activators: cell division cycle 20 (Cdc20) or Cdc20 homologue 1 (Cdh1, or Fizzy-related 1(Fzr1)).

These two co-activators differ in many aspects. First, they exert their functions at different mitotic stages (23). APC/C associates with Cdc20 during early mitosis to degrade substrates that function during prometaphase and metaphase. Subsequently, Cdh1 replaces Cdc20 during anaphase and G_1_ phase and mediates subsequent protein degradation events. By interacting with different co-activators, the APC/C complex promotes key cell cycle transitions. Second, Cdc20 and Cdh1 interact with their substrates *via* short recognition motifs or degrons (25). Cdc20 binds the Destruction box (D-Box): RXXLXXI/VXN, and Cdh1 binds the Lys-Glu-Asp (KEN) box. These targeting motifs ensure specific binding to the WD40 domain of Cdc20 or Cdh1, so that their timing of mitotic destruction is fine-tuned. Further single-molecule kinetics analysis revealed that the weak affinity with the D/Ken-Box motifs are complemented by priming ubiquitination events on the substrates, which enhances binding with APC/C. And subsequent processive affinity amplification will iteratively increase ubiquitin conjugation on the APC/C substrates to achieve both specificity and efficiency (26). Third, they are expressed in different tissues: Cdc20 is expressed strictly in proliferative tissues, but APC subunits and Cdh1 are also expressed in terminally differentiated cells, such as the adult brain (27).

In this article, we explored the underlying mechanism of OGT downregulation during mitosis. As it is known that OGT is most abundant in the brain (4), where Cdc20 is absent (27), we made an educated guess that mitotic degradation of OGT is probably mediated by APC/C^Cdc20^. By mutagenesis screening, we identified the D-box on OGT to be Arg-351/Leu-354. Using mass spectrometry, we mapped a potential priming ubiquitination site to be Lys-352. We further utilized mouse xenograft experiments to demonstrate that the R351C mutant and D box mutants suppressed uterine carcinoma progression, probably due to impaired cell proliferation. Our work thus reveals a key mechanism of how the cell cycle modulates the abundance of OGT and suggests that OGT is regulated by different biological stress and stimuli.

## Results

### OGT interacts with Cdc20

**Figure 1:**
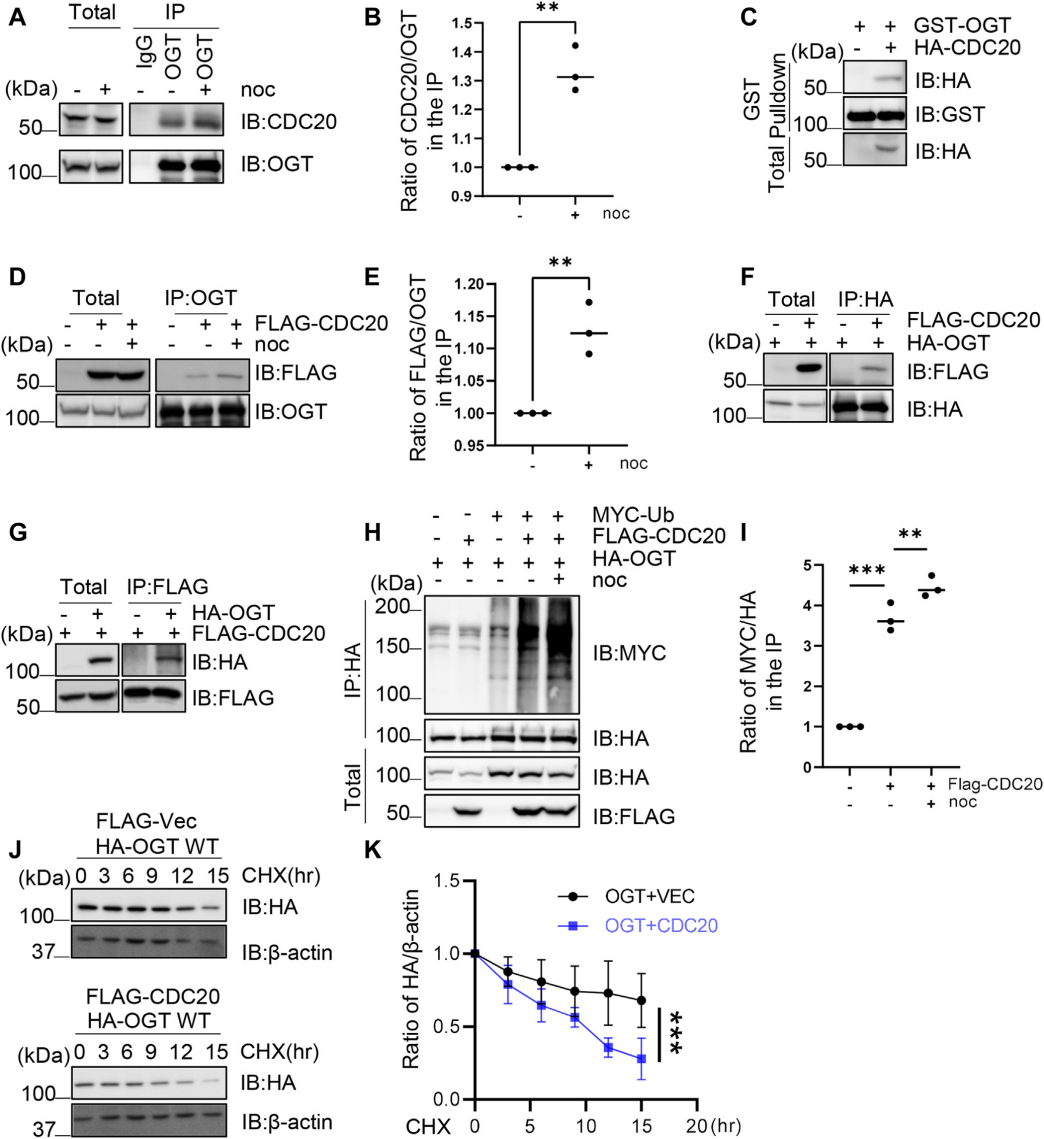
**CDC20 interacts with OGT and mediates OGT degradation.***A* and *B*, endogenous CDC20 and OGT co-immunoprecipitate. 293T cell were treated with Nocodazol (noc) or not, the lysates were immunoprecipitated with an anti-OGT antibody and immunoblotted with anti-OGT and anti-CDC20 antibodies. *C*, cells were transfected with HA-CDC20 plasmids, and the cell lysates were incubated with recombinant GST-OGT proteins. *D* and *E*, cells were transfected with Flag-Cdc20 plasmids and treated with noc or not. The cell lysates were subject to immunoprecipitation and immunoblotting with the antibodies indicated. *F* and *G*, exogenous HA-OGT and Flag-CDC20 co-immunoprecipitate reciprocally. Cells were transfected with HA-OGT and Flag-CDC20 plasmids, and the lysates were subject to immunoprecipitation and immunoblotting with the antibodies indicated. *H* and *I*, cells were transfected with Flag-CDC20, HA-OGT, and Myc-Ub (Myc-Ubiquitin), treated with noc or not, and the lysates were immunoprecipitated with an anti-HA antibody and immunoblotted with antibodies indicated. *J* and *K*, 293T cells were transfected with HA-OGT plasmids together with Flag-Vec or Flag-CDC20 plasmids, then cycloheximide (CHX) was added to block new protein synthesis. Quantitation in (*B*, *E*, and *I*) was carried out with a *t* test, in *K* was done with a two-way Anova. ∗∗ indicates *p* < 0.005; ∗∗∗ indicates *p* < 0.001. All western blots were repeated at least three times.

We are interested in exploring the mechanism of OGT downregulation during mitosis. Previous investigations have shown that OGT is most abundant in the brain tissues (4), where Cdh1 is present but Cdc20 is absent (27), suggesting that APC/C^Cdc20^ may degrade OGT. To test the possibility, we examined the interaction between Cdc20 and OGT. Cellulate lysates were first immunoprecipitated (IPed) with anti-OGT antibodies, and Cdc20 is present in the immunoprecipitates (Fig. 1*A*). We also used nocodazole (noc) to synchronize the cells, and then we observed an increase of OGT-Cdc20 interaction (Fig. 1, *A* and *B*). Recombinant GST-OGT proteins were used in pull-down assays, and GST-OGT pulled-down Cdc20 (Fig. 1*C*). We then assessed the association between overproduced Cdc20 and endogenous OGT (Fig. 1, *D* and *E*). Flag-Cdc20 coIPs with endogenous OGT, and the interaction becomes more robust after noc treatment (Fig. 1, *D* and *E*). Then interaction between exogenous Cdc20 and OGT was also tested (Fig. 1, *F* and *G*), and the immunoprecipitation experiments revealed reciprocal coIP between over-produced proteins. These results suggest that OGT interacts with Cdc20.

Then we evaluated whether Cdc20 modulates OGT stability. Cells were transfected with Flag-Cdc20, HA-OGT and Myc-Ub plasmids, and Cdc20 overproduction significantly increased the ubiquitination levels of OGT, and noc treatment further elevated OGT ubiquitination (Fig. 1, *H* and *I*). Cycloheximide (CHX) pulse-chase experiments were carried out (Fig. 1, *J* and *K*), and Cdc20 overproduction attenuated OGT stability, suggesting that Cdc20 degrades OGT during mitosis.

### Arg-351/Leu-354 of OGT is the D-box essential for Cdc20 interaction

**Figure 2:**
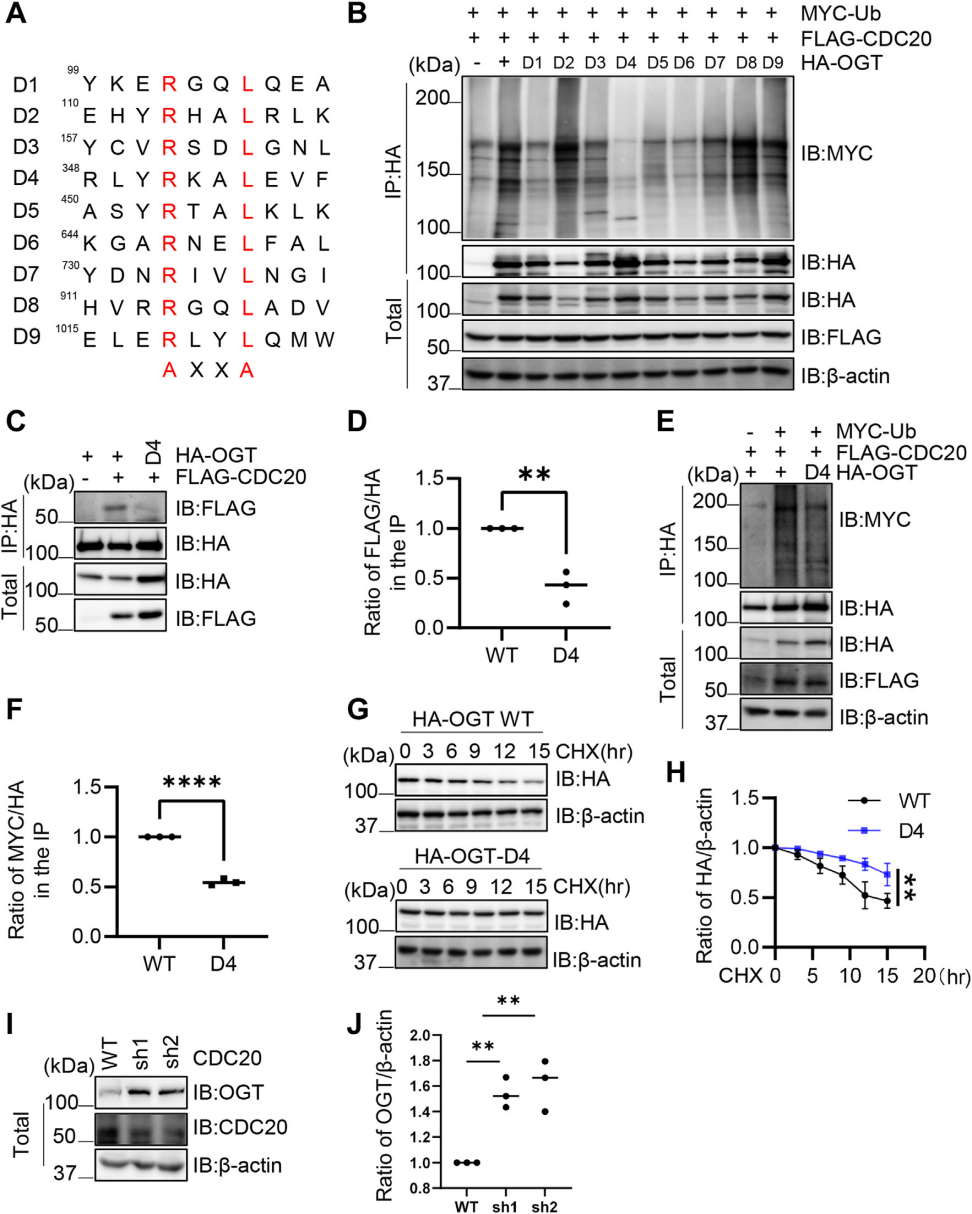
**APC/C**^**Cdc20**^**-dependent degradation of OGT.***A*, all potential D-box motifs in the OGT sequence were mutated. *B*, 293T cells were transfected with Myc-Ub, Flag-CDC20, HA-OGT-WT or D-box mutants, and the lysates were subject to immunoprecipitation and immunoblotting assays as indicated. *C* and *D*, 293T cells were transfected with HA-OGT-WT and -D4 plasmids, together with Flag-CDC20, and the lysates were subject to immunoprecipitation and immunoblotting with the antibodies indicated. *E* and *F*, 293T cells were transfected with Myc-Ub, Flag-CDC20 plasmids together with HA-OGT-WT or -D4 plasmids, then the anti-HA immunoprecipitates were immunoblotted with anti-Myc antibodies to detect the ubiquitination levels. *G* and *H*, 293T cells were transfected with HA-OGT-WT or -D4 plasmids, then cycloheximide (CHX) was added to block new protein synthesis. *I* and *J*, cells were transfected with two independent shRNAs targeting *CDC20*, and the lysates were collected and immunoblotted with the antibodies indicated. Quantitation in (*D*, *F*, and *I*) was done with a Student’s *t* test, in *H* was done with a two-way Anova. ∗∗ indicates *p* < 0.005; ∗∗∗∗indicates *p* < 0.0001. All western blots were repeated at least three times.

To find out the potential D-box of OGT, we did a mutagenesis screen by mutating all RXXL motifs (Fig. 2*A*). There are 9 RXXL motifs in OGT, and we named them as D1-D9. Then we used the nine mutants and tested their ubiquitination levels in cells (Fig. 2*B*), and D4 showed the least ubiquitination levels. Upon transfection of Cdc20, the ubiquitination levels of D4 mutants decreased about 50% (Fig. 2, *C* and *D*), probably due to reduced interaction with Cdc20 (Fig. 2, *E* and *F*). Then the stability of OGT-D4 was assessed with CHX pulse-chase experiments, and D4 showed increased abundance (Fig. 2, *G* and *H*), as expected. To validate that Cdc20 indeed affects OGT stability, we used two independent shRNAs targeting *CDC20*. As shown in Figure 2*I*, the two shRNAs effectively knocked-down Cdc20 protein levels. And OGT abundance was significantly enhanced (Fig. 2, *I* and *J*). These results suggest that R351/L354 is the D-box for Cdc20 binding and subsequent OGT degradation.

### OGT Lys-352 ubiquitination primes binding with APC/C

**Figure 3:**
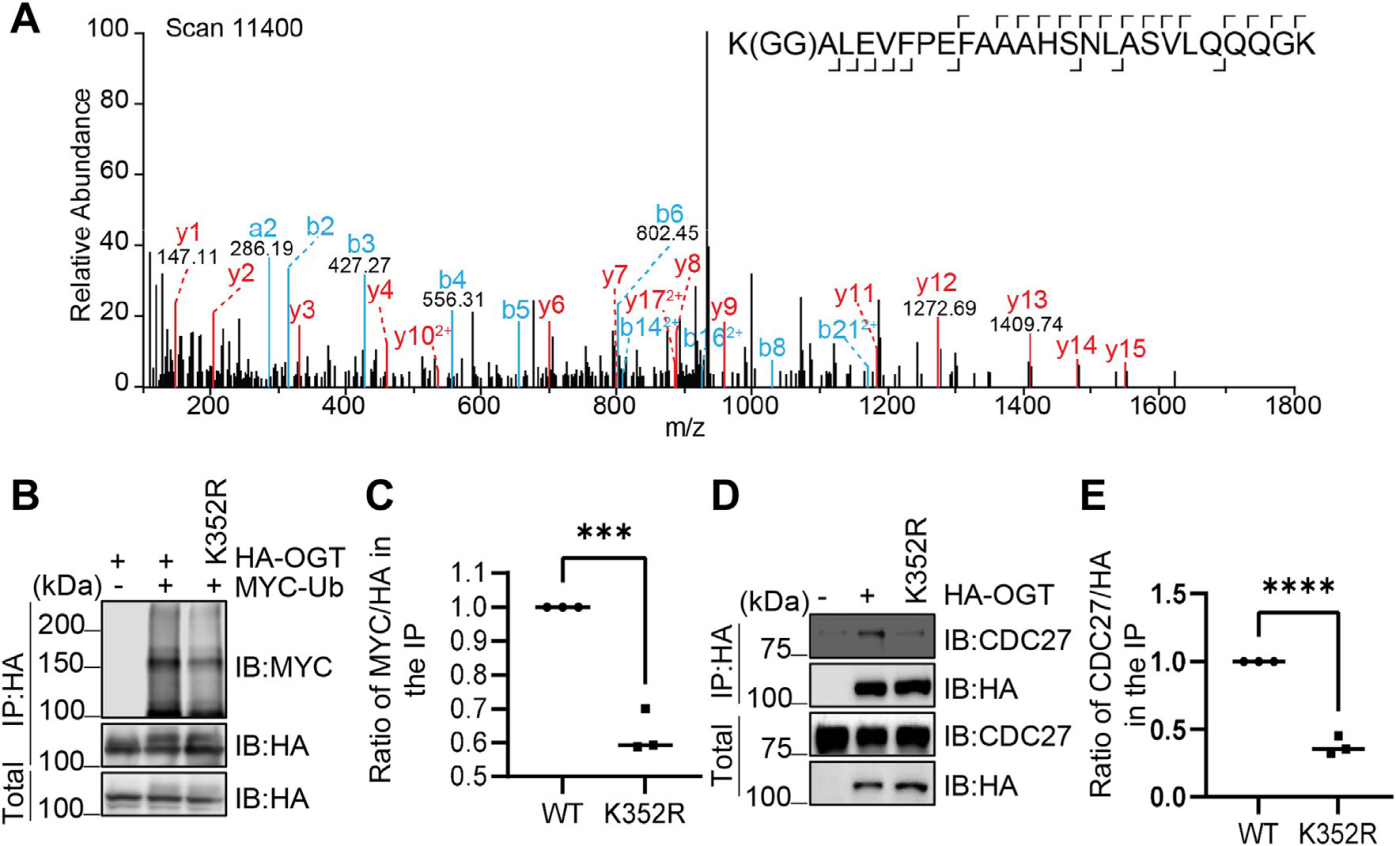
**K352 Ubiquitination promotes OGT-APC/C**^**CDC20**^**interaction.***A*, mass spectrometry revealed that Lys352 of OGT could be a ubiquitination site. *B* and *C*, 293T cells were transfected with HA-OGT-WT or -K352R plasmids, together with Myc-Ub, and the lysates were subject to immunoprecipitation and immunoblotting assays as indicated. *D* and *E*, 293T cells were transfected with HA-OGT-WT or -K352R plasmids, and the lysates were immunoprecipitated with an anti-HA antibody and immunoblotted with an anti-CDC27 antibody. Quantitation in *C* and *E* was done with a Student’s *t* test. ∗∗∗indicates *p* < 0.001; ∗∗∗∗indicates *p* < 0.0001. All western blots were repeated at least three times.

As APC/C-substrate binding often entails priming ubiquitination on the substrates (26), we used mass spectrometry (MS) to look for the potential priming ubiquitination site. During sample preparation, we transfected OGT and ubiquitin plasmids together and also used MG132 to block the subsequent proteasome-mediated degradation. Interestingly, MS revealed that Lys-352 is a proximal ubiquitination residue (Fig. 3*A*), neighboring the D box. We first verified the MS results by generating a K352R mutant and tested its ubiquitination, and K352R significantly decreased OGT ubiquitination (Fig. 3, *B* and *C*). The interaction between K352R and APC/C was further investigated by using the Cdc27 subunit of the APC/C complex (Fig. 3, *D* and *E*), and K352R markedly reduced binding with Cdc27. Taken together, OGT Lys-352 ubiquitination primes OGT binding with the APC/C complex.

### The TCGA R351C mutant increases OGT stability

**Figure 4:**
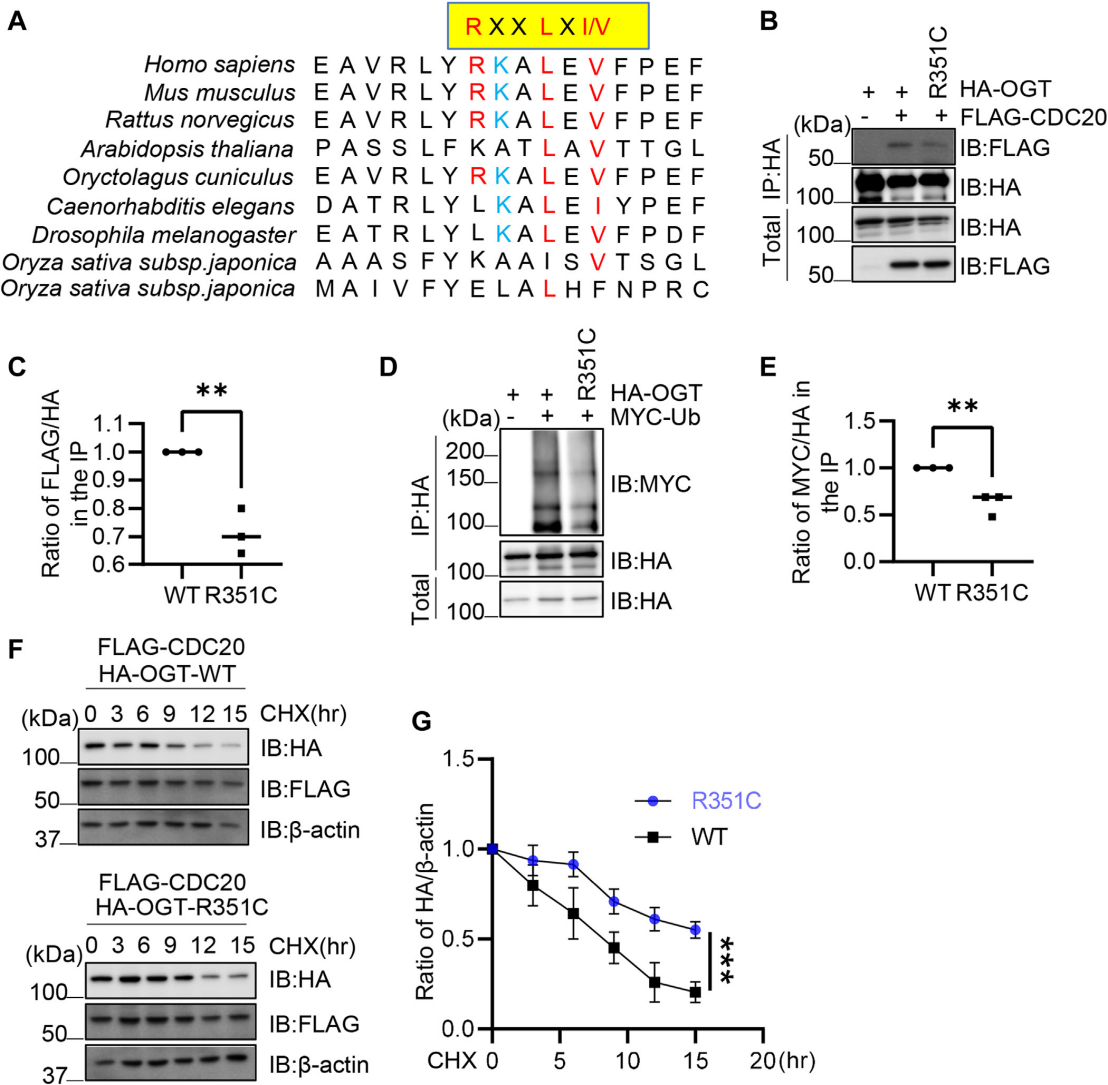
**R351C enhances the stability of OGT by decreasing CDC20-OGT binding.***A*, conservation of the D-box R351/L354 in different species. *B* and *C*, 293T cells were transfected with HA-OGT-WT or -R351C plasmids, together with Flag-CDC20, and the lysates were subject to immunoprecipitation and immunoblotting assays as indicated. *D* and *E*, 293T cells were transfected with HA-OGT-WT or -R351C plasmids, together with Myc-Ub. Then the anti-HA immunoprecipitates were immunoblotted with anti-Myc antibodies to detect the ubiquitination levels. *F* and *G*, 293T cells were transfected with HA-OGT-WT or -R351C plasmids, together with Flag-CDC20, then cycloheximide (CHX) was added to block new protein synthesis. Quantitation in *C* and *E* was done with a Student’s *t* test, in *G* was done with a two-way Anova. ∗∗indicates *p* < 0.005; ∗∗∗indicates *p* < 0.001. All western blots were repeated at least three times.

In TCGA, the R351C mutant is associated with uterine carcinoma, suggesting that the defects in the D box may lead to human diseases. Therefore we studied the R351C mutant. As shown in Figure 4*A*, the Arg-351 residue is conserved in mouse and rat, but not in the fly, worms, or plants. R351C decreased binding with Cdc20 (Fig. 4, *B* and *C*), reduced OGT ubiquitination (Fig. 4, *D* and *E*) and increased OGT stability (Fig. 4, *F* and *G*), which phenocopies the D box mutant. Taken together, the R351C mutant impaired the D-box function.

### The R351C and D-box mutants displayed mitotic defects

**Figure 5:**
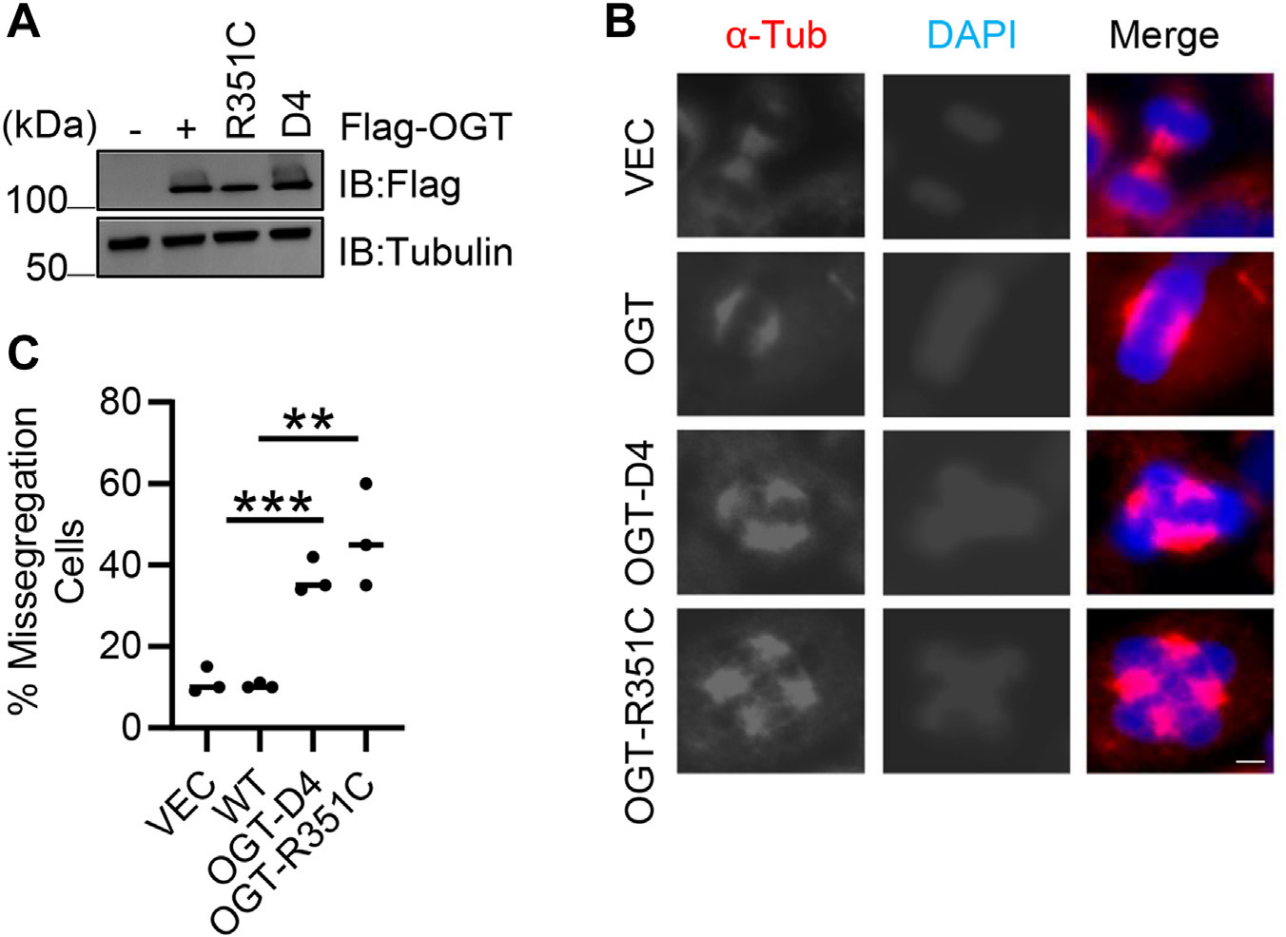
**Mitosis defects are observed in stable OGT-D4 and -R351C cell lines.***A*, HeLa cells stably expressing OGT-WT, -R351C and-D4 plasmids were constructed. *B* and *C*, the cells were subject to immunofluorescence analysis by staining with anti-α-tubulin antibodies and DAPI. Scale bar, 10 μm. ∗∗indicates *p* < 0.005; ∗∗∗indicates *p* < 0.001. Immunofluorescence experiments were repeated three times, with 100 cells per experiment.

As OGT is essential for faithful mitotic division (3, 6, 7), we wondered whether the R351C and D-box mutants would show mitotic defects. To this end, stable transfectants were generated that harbor the OGT-WT, -R351C, and -D4 mutants in HeLa cells (Fig. 5*A*), and mitosis was analyzed with immunofluorescence (Fig. 5, *B* and *C*). As expected, OGT D-box mutants displayed multinucleated cells, suggesting that mitotic degradation of OGT ensures a correct cell division.

### The R351C and D-box mutants suppress uterine carcinoma

**Figure 6:**
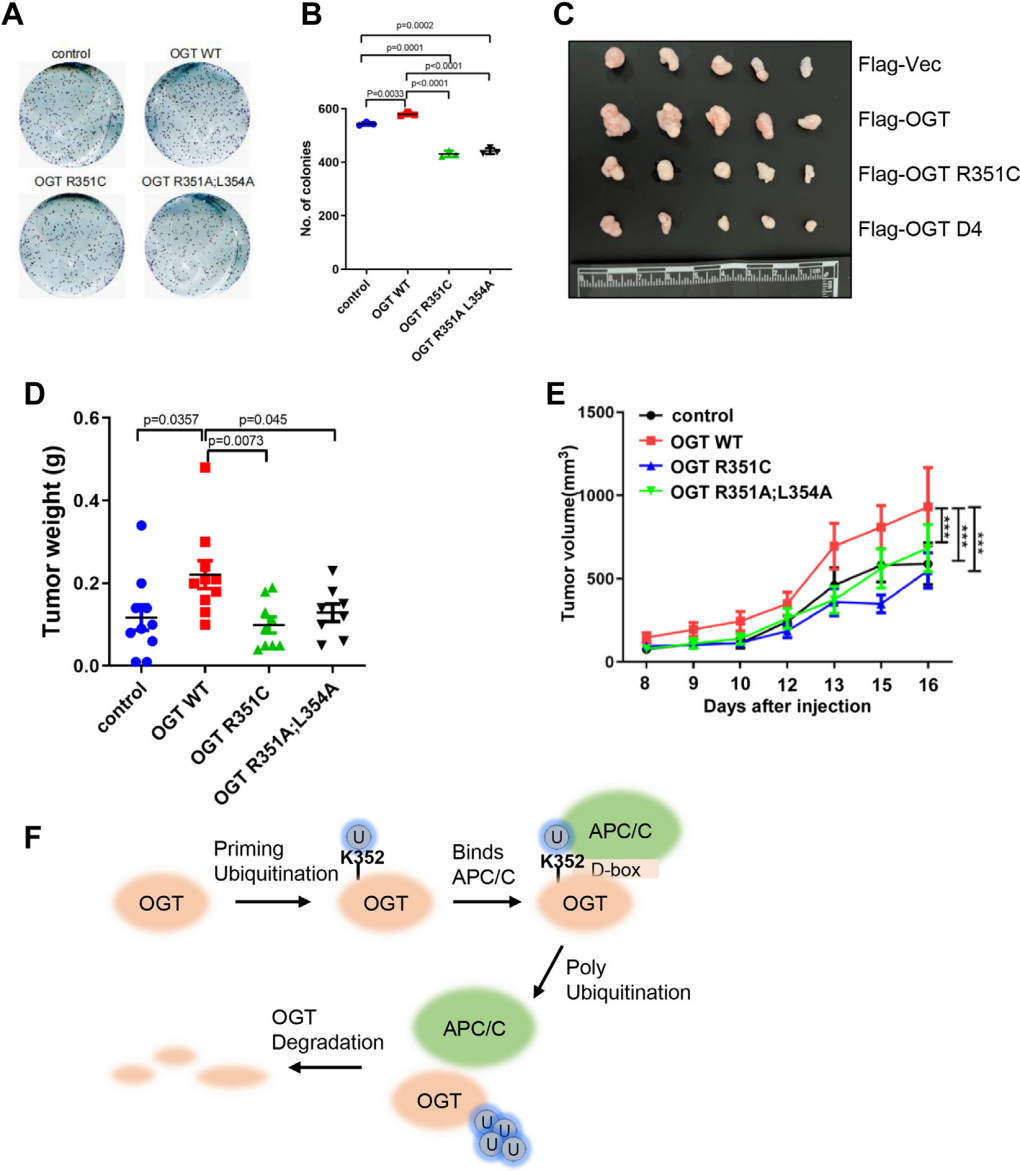
**The OGT D box mutant decreases uterine carcinoma in mouse xenograft models.***A* and *B*, colony formation assay showing that OGT-D4 and R351C mutants decrease cell proliferation. *C–E*, stable OGT-WT, -D4 and -R351C HeLa cells were injected into nude mice, and the tumors were photographed. Tumor weights were quantitated in (*D*), and tumor sizes were quantitated in (*E*). Quantitation was done with a one-way Anova. ∗*p*< 0.05; ∗∗*p*< 0.005. *F*, a model showing that APC/C^CDC20^ mediates degradation of OGT upon mitotic onset: Priming ubiquitination occurs on OGT-K352, which promotes OGT-APC/C^CDC20^ binding at the D-box motif; subsequently APC/C^CDC20^ ubiquitinates OGT and degrades OGT during mitosis.

Then we used mouse xenograft models to study the mutant effects *in vivo*. We first used colony formation assays, and the D box mutants decreased cell proliferation (Fig. 6, *A* and *B*), consistent with their mitotic defects as observed in Figure 5*B*. Then, the cells were implanted in nude mice, and Figure 6, *C*–*E* showed that smaller uterine carcinoma were evident in the mutants compared to OGT-WT. We reasoned that this may also be ascribed to the mitotic defects caused by the mutants.

## Discussion

In this work, we present evidence that OGT is under the regulation of the cell cycle machinery, specifically, the APC/C^Cdc20^ E3 ligase. We propose a model (Fig. 6*F*): OGT is first ubiquitinated at K352 to promote APC/C^Cdc20^-OGT binding, which enhances the affinity between APC/C^Cdc20^ and OGT *via* a conserved D box; subsequently, APC/C^Cdc20^ ubiquitinates OGT and subject it to degradation. Besides D-box, APC/C ^Cdc20^ substrates also have other degrons, such as GxEN box (28). Our study does not exclude the possibility that OGT may contain other potential degrons.

Our work is in line with the recent notion that short linear sequence motifs (SLIMs) modulate the interaction among APC/C, its substrates, and coactivators (29). Specifically, we found that the conserved Arg351/Leu354 D-box mediates the interaction between OGT and APC/C subunits. As SLIMs confer versatility and variability to protein-protein interaction during evolution (30), it is no surprise that the D-box only came into existence in mice, rats, and humans but not in other model organisms (Fig. 4*A*). We think that OGT may contain many such SLIMs that complex OGT with other important biological regulators.

It is not uncommon that the SLIMs are subject to other PTMs. As a subtype of SLIM, degrons are also often subject to PTMs (31, 32). For example, Set1, the writer for histone H3K4me3, is ubiquitinated by APC/C^Cdh1^ (33). Set1 also contains a D-box. When the D-box is phosphorylated by Cla4, Set1 proteolysis is prevented (33). Our work provides another example where ubiquitination within the OGT degron promotes OGT-APC affinity and subsequent OGT ubiquitination.

As a central E3 ligase, APC/C^Cdc20^ is subject to myriad PTMs (34): a total of 68 conserved mitotic phosphorylation sites were identified on APC/C bound to Cdc20, which modulates APC/C-Cdc20 binding and subsequent activation (34). Recently, lactate has been shown to inhibit SENP1 and stabilizes APC4 SUMOylation and UBE2C-APC/C binding, thus regulating the timing of APC/C function (35). Therefore, PTMs act synergistically with SLIMs for APC/C activation, substrate recognition, and subsequent proteolysis.

We observed the D box mutants suppress uterine carcinoma in mouse xenograft models, but in TCGA the mutants correlate with human uterine carcinoma events. We cannot resolve the discrepancy at this moment. It would be possible that the mutants are tumorigenic due to reasons not directly related to the cell cycle. In fact, the APC/C ubiquitin ligase has been implicated in cellular differentiation, by regulating proteins involved in fate determination in stem cells (36). As OGT also regulates the pluripotency network in mouse embryonic stem cells (37), the D-box mutants may have other functions in stem cells. In sum, our results suggest that cell division regulates OGT in many ways, and a deeper understanding of its biochemical mechanism will contribute to developing more interrogation chemical tools.

## Experimental procedures

### Cell culture, antibodies, and plasmids

HeLa and 293T cells were purchased from ATCC. The cell lines were validated using STR profiling and free from *mycoplasma* contamination for all experiments. Antibodies were as follows: anti-Myc (PTM Bio, #PTM-5390), anti-Flag (Sigma, F1084), anti-GST (Gene Script, A00865), anti-HA (Bethyl Laboratories, A190–108A), anti-OGT (Abcam, ab96718), anti-CDC20 (CST, #4823S), anti-CDC27 (ProteinTech, #10918-1-AP), and anti-β-actin (Sigma, A5441), anti-tubulin (MBL, PM054). OGT mutant plasmids were generated using specific primers (sequences available upon request) following the manufacturer's instructions (QuickChange II, Stratagene). shRNA sequences targeting *CDC20* are: AGACCAACCCATCACCTCAGT, TGGTGGTAATGATAACTTGGT.

### Immunoprecipitation and immunoblotting assays

Immunoprecipitation and immunoblotting experiments were performed as described before (38). The following primary antibodies were used for immunoblotting: anti-HA (1:5000), anti-FLAG M2 (Sigma) (1:1000), anti-OGT (1:1000), anti-Myc (1:3000), anti-GST (1:3000), anti-β-actin (1:3000), anti-CDC27 (1:2000), and anti-CDC20 (1:1000). Peroxidase-conjugated secondary antibodies were from JacksonImmuno Research. The ECL detection system (Amersham) was used for immunoblotting. LAS-4000 was employed to detect signals and quantitated using Multi Gauge software (Fujifilm). All Western blots were repeated at least three times.

### LC-MS/MS analysis

Cells were transfected with OGT and ubiquitin plasmids and also treated with MG132 to block proteasome-mediated degradation. The OGT proteins were immunoprecipitated and analyzed by SDS-PAGE. The gel was stained by Coomassie blue and then destained by a solution containing 30% ethanol and 12.5% acetic acid in H_2_O. The protein band of OGT was excised from the gel and ground into tiny pieces for the subsequent reduction and alkylation reactions. For protein digestion, dehydrated gel pieces were rehydrated with a trypsin solution (2 ng/μl) and incubated at 37 °C for 16 h. Next, the supernatant containing digested peptides was collected, and gel pieces were once again dehydrated using 75% Acetonitrile for 15 min at room temperature. The resulted supernatant was combined with the previous supernatant. Finally, the samples were dried and stored at −80 °C until MS analysis.

For MS analysis, dry peptides were dissolved in 0.1% formic acid in water and loaded to a Dionex Ultimate 3000 RPLC nano system coupled to a Q Executive Plus spectrometer (Thermo Fisher Scientific). Full scan MS spectra were acquired at a resolution of 70,000 with an auto gain control (AGC) target value of 3 × 10^6^ ions. The top 12 precursor peptides were fragmented by High-energy collisional dissociation (HCD) with a normalized collision energy of 28%. MS raw data were directly processed by MaxQuant 2.3.1.0 against the UniProt Human Reference Proteome (21,006 entries, downloaded in April 2016). The enzyme specificity was set as trypsin and the maximum number of missed cleavages was set to 2. Cysteine carbamidomethylation was set as a fixed modification. Methionine oxidation, protein N-terminal acetylation, and diglycine remnants of lysine were set as variable modifications. Default MaxQuant settings were used: the initial and mass tolerance were 20 ppm and 4.5 ppm, respectively, and the MS/MS match tolerance was 20 ppm. For the identification of modified peptides, the minimum Andromeda score was 40, the minimum delta score was 6. The result was filtered at 1% PSM-level false discovery rate (FDR) and 1% site-level FDR.

### Indirect immunofluorescence staining

Indirect immunofluorescence staining was carried out as described previously (39). Antibody dilutions were 1:1000 for mouse anti-α-tubulin. The nuclei were stained with DAPI. All immunofluorescence experiments were repeated three times, with 100 cells per experiment.

### Establishment of stable overexpressed cell lines

We used the lentivirus-based vector phage-flag-vet obtained from Genechem (Shanghai, China), phage-Flag-vet-OGT-WT; OGT-R351C; OGT-R351C, L354A plasmids were constructed, which were used then to infect the HeLa cell lines. We screened out the stable cell lines with puromycin (2 μg/ml).

### Colony formation assay

The stable cell lines were collected and then inoculated into six-well plates at a density of 500 cells per well. After 2 weeks, cells were washed with PBS, fixed with polymethanol, and stained with 1 ml 0.1% Crystal violet. And the colonies were counted.

### Mouse xenograft analysis

Approximately 1 × 10^6^ HeLa cells (phage-FLAG-vec; OGT-WT; OGT-R351C; OGT-R351C， L354A cell lines) per mouse suspended in 100 μl PBS were injected in the flank of male BALB/c nude mice (4 weeks old). During the 14th day observation, the tumor size (V = (width2 × length × 0.52)) was measured with vernier caliper. Mice were anesthetized and then sacrificed, and the tumors were separated and weighed and photographed. The mice were obtained from the Animal Research and Resource Center, Yunnan University (Certification NO. SCXK[Dian]K2021–0001). All animal studies and manipulations were performed in compliance with the institutional guidelines approved by the animal research and resource center, Yunnan University (Kunming, China).

## Data availability

The MS proteomics data have been deposited to the ProteomeXchange Consortium *via* the PRIDE partner repository with the dataset identifier PXD050552. The spectrum in Figure 3*A* was selected from the best PEP scan number according to MaxQuant results.

## Conflict of interest

The authors declare that they have no known competing financial interests or personal relationships that could have appeared to influence the work reported in this paper.

## References

[1] Hart G.W., Slawson C., Ramirez-Correa G., Lagerlof O. (2011). Cross talk between O-GlcNAcylation and phosphorylation: roles in signaling, transcription, and chronic disease. Annu. Rev. Biochem..

[2] Yang X., Qian K. (2017). Protein O-GlcNAcylation: emerging mechanisms and functions. Nat. Rev. Mol. Cell Biol..

[3] Levine Z.G., Walker S. (2016). The biochemistry of O-GlcNAc transferase: which functions make it essential in mammalian cells?. Annu. Rev. Biochem..

[4] Wang Z., Udeshi N.D., Slawson C., Compton P.D., Sakabe K., Cheung W.D. (2010). Extensive crosstalk between O-GlcNAcylation and phosphorylation regulates cytokinesis. Sci. Signal..

[5] Tan E.P., Duncan F.E., Slawson C. (2017). The sweet side of the cell cycle. Biochem. Soc. Trans..

[6] Liu C., Li J. (2018). O-GlcNAc: a sweetheart of the cell cycle and DNA damage response. Front. Endocrinol. (Lausanne).

[7] Saunders H., Dias W.B., Slawson C. (2023). Growing and dividing: how O-GlcNAcylation leads the way. J. Biol. Chem..

[8] Magescas J., Sengmanivong L., Viau A., Mayeux A., Dang T., Burtin M. (2017). Spindle pole cohesion requires glycosylation-mediated localization of NuMA. Sci. Rep..

[9] Li Q., Kamemura K. (2014). Adipogenesis stimulates the nuclear localization of EWS with an increase in its O-GlcNAc glycosylation in 3T3-L1 cells. Biochem. Biophys. Res. Commun..

[10] Lanza C., Tan E.P., Zhang Z., Machacek M., Brinker A.E., Azuma M. (2016). Reduced O-GlcNAcase expression promotes mitotic errors and spindle defects. Cell Cycle.

[11] Liu C., Shi Y., Li J., Liu X., Xiahou Z., Tan Z. (2020). O-GlcNAcylation of myosin phosphatase targeting subunit 1 (MYPT1) dictates timely disjunction of centrosomes. J. Biol. Chem..

[12] Yuan A., Tang X., Li J. (2020). Centrosomes: Til O-GlcNAc do us apart. Front. Endocrinol. (Lausanne).

[13] Yan S., Peng B., Kan S., Shao G., Xiahou Z., Tang X. (2023). Polo-like kinase 1 (PLK1) O-GlcNAcylation is essential for dividing mammalian cells and inhibits uterine carcinoma. J. Biol. Chem..

[14] Li Z., Li X., Nai S., Geng Q., Liao J., Xu X. (2017). Checkpoint kinase 1-induced phosphorylation of O-linked beta-N-acetylglucosamine transferase regulates the intermediate filament network during cytokinesis. J. Biol. Chem..

[15] Slawson C., Lakshmanan T., Knapp S., Hart G.W. (2008). A mitotic GlcNAcylation/phosphorylation signaling complex alters the posttranslational state of the cytoskeletal protein vimentin. Mol. Biol. Cell.

[16] Komura K., Ise H., Akaike T. (2012). Dynamic behaviors of vimentin induced by interaction with GlcNAc molecules. Glycobiology.

[17] Slawson C., Zachara N.E., Vosseller K., Cheung W.D., Lane M.D., Hart G.W. (2005). Perturbations in O-linked beta-N-acetylglucosamine protein modification cause severe defects in mitotic progression and cytokinesis. J. Biol. Chem..

[18] Sakabe K., Hart G.W. (2010). O-GlcNAc transferase regulates mitotic chromatin dynamics. J. Biol. Chem..

[19] Ruan H.B., Ma Y., Torres S., Zhang B., Feriod C., Heck R.M. (2017). Calcium-dependent O-GlcNAc signaling drives liver autophagy in adaptation to starvation. Genes Dev..

[20] Na H.J., Akan I., Abramowitz L.K., Hanover J.A. (2020). Nutrient-driven O-GlcNAcylation controls DNA damage repair signaling and stem/progenitor cell homeostasis. Cell Rep..

[21] Stephen H.M., Adams T.M., Wells L. (2021). Regulating the regulators: mechanisms of substrate selection of the O-GlcNAc cycling enzymes OGT and OGA. Glycobiology.

[22] Nakayama K.I., Nakayama K. (2006). Ubiquitin ligases: cell-cycle control and cancer. Nat. Rev. Cancer.

[23] Sivakumar S., Gorbsky G.J. (2015). Spatiotemporal regulation of the anaphase-promoting complex in mitosis. Nat. Rev. Mol. Cell Biol..

[24] Watson E.R., Brown N.G., Peters J.M., Stark H., Schulman B.A. (2019). Posing the APC/C E3 ubiquitin ligase to orchestrate cell division. Trends Cell Biol..

[25] Primorac I., Musacchio A. (2013). Panta rhei: the APC/C at steady state. J. Cell Biol..

[26] Lu Y., Lee B.H., King R.W., Finley D., Kirschner M.W. (2015). Substrate degradation by the proteasome: a single-molecule kinetic analysis. Science.

[27] Gieffers C., Peters B.H., Kramer E.R., Dotti C.G., Peters J.M. (1999). Expression of the CDH1-associated form of the anaphase-promoting complex in postmitotic neurons. Proc. Natl. Acad. Sci. U. S. A..

[28] Tan G.S., Lewandowski R., Mallory M.J., Strich R., Cooper K.F. (2013). Mutually dependent degradation of Ama1p and Cdc20p terminates APC/C ubiquitin ligase activity at the completion of meiotic development in yeast. Cell Div..

[29] Barford D. (2020). Structural interconversions of the anaphase-promoting complex/cyclosome (APC/C) regulate cell cycle transitions. Curr. Opin. Struct. Biol..

[30] Tompa P., Davey N.E., Gibson T.J., Babu M.M. (2014). A million peptide motifs for the molecular biologist. Mol. Cell.

[31] Lee J.M., Hammaren H.M., Savitski M.M., Baek S.H. (2023). Control of protein stability by post-translational modifications. Nat. Commun..

[32] Meszaros B., Kumar M., Gibson T.J., Uyar B., Dosztanyi Z. (2017). Degrons in cancer. Sci. Signal..

[33] Gong X., Wang S., Yu Q., Wang M., Ge F., Li S. (2023). Cla4 phosphorylates histone methyltransferase Set1 to prevent its degradation by the APC/C(Cdh1) complex. Sci. Adv..

[34] Qiao R., Weissmann F., Yamaguchi M., Brown N.G., VanderLinden R., Imre R. (2016). Mechanism of APC/CCDC20 activation by mitotic phosphorylation. Proc. Natl. Acad. Sci. U. S. A..

[35] Liu W., Wang Y., Bozi L.H.M., Fischer P.D., Jedrychowski M.P., Xiao H. (2023). Lactate regulates cell cycle by remodelling the anaphase promoting complex. Nature.

[36] Kimata Y. (2019). APC/C ubiquitin ligase: coupling cellular differentiation to G1/G0 phase in multicellular systems. Trends Cell Biol..

[37] Hao Y., Li X., Qin K., Shi Y., He Y., Zhang C. (2023). Chemoproteomic and transcriptomic analysis reveals that O-GlcNAc regulates mouse embryonic stem cell fate through the pluripotency network. Angew. Chem. Int. Ed. Engl..

[38] Li J., Wang J., Hou W., Jing Z., Tian C., Han Y. (2011). Phosphorylation of Ataxin-10 by polo-like kinase 1 is required for cytokinesis. Cell Cycle.

[39] Xu X., Stern D.F. (2003). NFBD1/KIAA0170 is a chromatin-associated protein involved in DNA damage signaling pathways. J. Biol. Chem..

